# Plasmofluidic Microlenses for Label-Free Optical Sorting of Exosomes

**DOI:** 10.1038/s41598-019-44801-3

**Published:** 2019-06-13

**Authors:** Xiangchao Zhu, Ahmet Cicek, Yixiang Li, Ahmet Ali Yanik

**Affiliations:** 1 Department of Electrical and Computer Engineering, University of California, Santa Cruz, CA 95064 USA; 2 Department of Nanoscience and Nanotechnology, Burdur Mehmet Akif Ersoy University, Burdur, 15030 Turkey; 3 California Institute for Quantitative Biosciences (QB3), University of California, Santa Cruz, CA 95064 USA

**Keywords:** Optofluidics, Microfluidics, Nanophotonics and plasmonics

## Abstract

Optical chromatography is a powerful optofluidic technique enabling label-free fractionation of microscopic bioparticles from heterogenous mixtures. However, sophisticated instrumentation requirements for precise alignment of optical scattering and fluidic drag forces is a fundamental shortcoming of this technique. Here, we introduce a subwavelength thick (<200 nm) Optofluidic PlasmonIC (OPtIC) microlens that effortlessly achieves objective-free focusing and self-alignment of opposing optical scattering and fluidic drag forces for selective separation of exosome size bioparticles. Our optofluidic microlens provides a self-collimating mechanism for particle trajectories with a spatial dispersion that is inherently minimized by the optical gradient and radial fluidic drag forces working together to align the particles along the optical axis. We demonstrate that this facile platform facilitates complete separation of small size bioparticles (i.e., exosomes) from a heterogenous mixture through negative depletion and provides a robust selective separation capability for same size nanoparticles based on their differences in chemical composition. Unlike existing optical chromatography techniques that require complicated instrumentation (lasers, objectives and precise alignment stages), our OPtIC microlenses with a foot-print of 4 μm × 4 μm open up the possibility of multiplexed and high-throughput sorting of nanoparticles on a chip using low-cost broadband light sources.

## Introduction

Optical chromatography is a powerful fractionation technique that has recently gained significant attention for label-free separation and analysis of microscopic bioparticles (cells, bacteria, etc.)^1–4^. This technique relies on a mildly focused Gaussian laser beam along a microfluidic channel to create opposing optical scattering and fluidic drag forces^5,6^. One can harness the varying strength and balance of these forces on bioparticle size, composition and morphology for selective sorting. Initially demonstrated for size-based elution of polystyrene beads, optical chromatography has been successfully applied to fractionation of blood components such as human erithrocytes, monocytes, granulocytes, and lymphocytes^2,5^. This technique particularly excels in distinguishing bioparticles with subtle differences^4–7^. Its highly precise separation capability has been recently demonstrated using micron size bioparticles with diameters that differ by less than 70 nm^8^. Most remarkably, separation of two closely related genetic relatives, *Bacillus anthracis* and *Bacillus thuringiensis*^4^, and cells with single gene modifications^3^ has been achieved using small differences in their chemical makeup (refractive index). In essence, optical chromatography offers unique capabilities as a modern separation technique, especially when combined with multi-stage sequential fractionation and microfluidic network-based purification approaches^9,10^. However, several critical limitations must be overcome for its wide adaptation: (1) To create strong optical scattering forces along the microfluidic channels, high cost laser sources are needed^2^. (2) Laser beam must be precisely aligned along the fluidic channel with a well-controlled beam waist profile, requiring a complicated optical alignment procedure that employs multiple multi-axis positioners^5^. (3) Scaling of existing optical chromatography techniques for multiplexed and high throughput operation is not practical, since each channel requires separate alignment sensitive and high-cost laser sources and objectives^10^.

In this report, we introduce **O**ptofluidic **P**lasmon**IC (OPtIC)** microlenses to overcome the shortcomings of existing optical chromatography techniques by eliminating the need for sophisticated instrumentation and precise alignment requirements. Our sub-wavelength thick (~200 nm thick) OPtIC microlenses offer objective-free focusing and self-alignment of optical and fluidic drag forces and present a facile platform for selective separation of exosome size bioparticles. By allowing direct coupling of collimated broadband light to realize strong optical scattering forces at a focal point, extremely small footprint (4 μm × 4 μm) OPtIC microlenses open the door for drastically multiplexed optical chromatography and high-throughput sample processing capability. In addition to parallel operation, laterally integrated OPtIC microlenses on a planar chip permit serial microfluidic schemes^9,10^ to be readily implemented for multi-stage sequential separation and purification using broadband light sources and conventional planar microfluidic approaches. In the following, we show that our optofluidic sorting scheme based on OPtIC microlenses enables selective separation of exosome size bioparticles (<200 nm in diameter). Furthermore, we demonstrate that our platform offers readily tunable, highly reliable and selective separation of nano-bioparticles by adjusting the light intensity (i.e. radiation pressure) and/or the fluid flow rate (i.e. opposing drag force) based on size and minor difference in chemical makeup (refractive index).

## Results

### Optofluidic-plasmonic (OPtIC) microlenses

**Figure 1:**
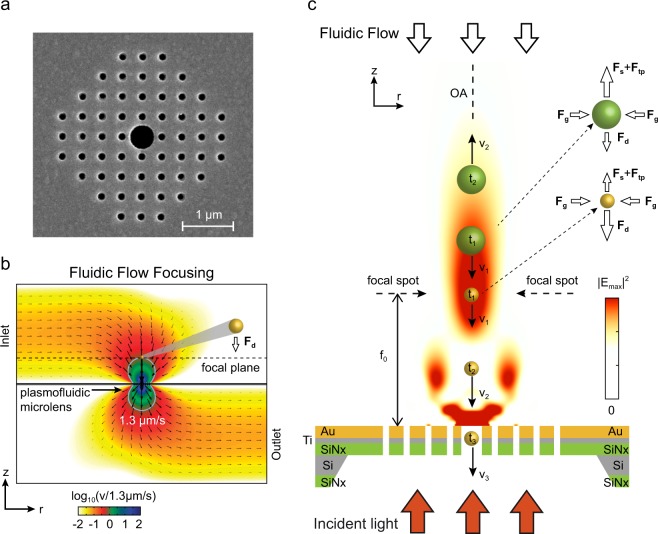
(**a**) A 4 μm × 4 μm OPtIC microlens consisting of a patch array of small circular nanoholes and an enlarged center aperture for nanofluidic integration. (**b**) Fluidic flow profile through the OPtIC microlens with a fluidic flow rate of 1.3 μm/s at the focal point. (**c**) Separation of two different size particle is illustrated at the focal point region. Fluidic drag (**F**_**d**_), optical scattering (**F**_**s**_) and thermo-plasmonic convection (**F**_**tp**_) forces are inherently aligned against each other along the optical axis by the OPtIC microlens.

As shown in recent studies, periodic and quasi-periodic arrays of plasmonic nanoapertures can be used as micro-convex lenses focusing incident plane waves to dimensions comparable to optical wavelengths^11–13^. In addition to significantly-small fingerprints, this focusing capability can be exploited to realize large optical scattering forces using collimated broadband light sources^14,15^. In particular, finite size nanole array (NHA) structures provide a broadband focusing capability that is mainly controlled by the overall size of the array and tolerant of substructural variations^13^. The distinct nature of light focusing mechanism in NHA microlenses opens the door for nanofluidic integration through alterations in array design without degrading their focusing characteristics. A suspended plasmofluidic (OPtIC) microlens based on this principle is shown in Fig. 1. Here, an 9 × 9 array of nanoholes with a diameter of *d* = 150 nm and a periodicity of *a*_0_ = 380 nm is considered (Fig. 1a). NHA patch with a foot print of 4 μm × 4 μm is defined through a 120 nm thick gold film, 10 nm thick accompanying titanium (Ti) adhesion layer and a suspended silicon nitride (Si_3_N_4_) membrane (thickness of 100 nm) for microfluidic access from both side of it^16^. The periodic nanohole lattice allows enhanced light transmission through extraordinary light transmission effect (EOT)^17–20^. The center nanohole is enlarged to facilitate nanofluidic flow. In Fig. 1a, the enlarged center hole diameter is *d*_*c*_ = 500 nm. The overall device is a hybrid nanoplasmonic and nanofluidic system in which fluidic flow through the enlarged center aperture is achieved by pumping a solution from an inlet port on the top side and extracting it through an outlet port on the bottom side^21,22^. The inlet and outlet ports are located away from the plasmofluidic microlense to provide a clear path for the optical beam^21^. Following Hagen-Poiseille’s law *Q* = *Δp/R*_*H*_ [m^3^ s^−1^], the pressure-driven flow through a circular opening of length *h* can be understood using hydraulic resistance *R*_*H*_, which is inversely proportional to the fourth power of the opening radius^23^: ${R}_{H}\approx {8\,\mu h/\pi r}_{H}^{4}$ [Pa s^3^ m^−1^], where *μ* = 8.9 × 10^−4^ Pa·s is the dynamic viscosity of water and *h* = *t*_*Au*_ + *t*_*Ti*_ + *t*_*SiN*_ = 205 nm is the cylindrical conduit thickness. For an enlarged circular center aperture of *d*_*c*_ = 500 nm, the hydraulic resistance is more than two orders of magnitude smaller than that of the smaller nanoholes (*d* = 150 nm) around it. The least fluidic resistance path through the enlarged center aperture (*d*_*c*_ = *5*00 nm) leads to focusing of the convective fluidic flow along optical axis (OA), as demonstrated in our finite-element method (FEM) microfluidic calculations (Fig. 1b). Here, the flow through the centrally located enlarged aperture serves two purposes: (1) it brings nanoparticles to the focal point of the OPtIC microlense, and (2) it forces nanoparticles to follow a trajectory that goes through the OA, as illustrated in Fig. 1c. One can achieve optofluidic alignment by introducing a collimated light beam that is perpendicularly incident from the bottom of the OPtIC microlens (Fig. 1c). The beam focused by the OPtIC microlens, hence optical scattering force (**F**_**s**_), is spontaneously aligned against the fluidic drag force (**F**_**g**_) along the OA. Electromagnetic heating of the plasmofluidic microlense establishes a temperature gradient and heat induced convection current. The resulting thermo-plasmonic drag force (**F**_**tp**_) contributes to the optical scattering processes in balancing the fludic drag. As illustrated in Fig. 1c., in addition to providing a scattering force (**F**_**s**_) along the OA, light focused through the OPtIC microlens introduces an optical gradient force (**F**_**g**_) that radially pushes particles towards the OA. Combined with the radial drag forces (**F**_**d,r**_), at the focal point region, optical gradient force provides a robust mechanism for precise alignment of particles along the OA, as illustrated in Fig. 1c. For sufficiently large optical pressures that overcome the fluidic drag force (F_s_ > F_d_), particles are propelled against the fluidic flow along the OA. Hence, larger and higher refractive index particles (Fig. 1c, green color) experiencing larger optical scattering forces are kept at a distance away from the planar surface, whereas the smaller diameter and lower refractive index particles (Fig. 1c, yellow colored) are allowed to pass through the enlarged center aperture, resulting in complete separation of small size bioparticles (i.e., exosomes) from a mixture of larger size particles through negative depletion. Here, the OPtIC microlens serves as a far-field screen that prevents clogging of the center nanofluidic channel by keeping larger bioparticles away from the nanoaperture surface and high flow rate regions close to the center aperture.

### Light focusing with plasmofluidic microlens

**Figure 2:**
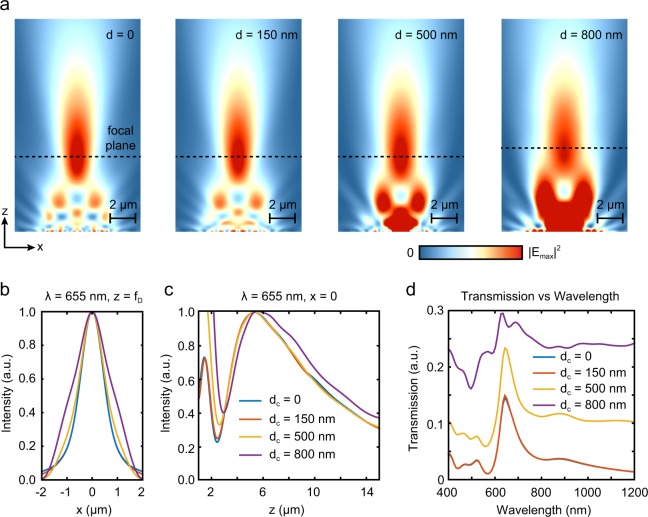
(**a**) Focused beam profiles are shown for OPtIC microlenses with varying center aperture diameter at *λ* = 655 nm. (**b**) Field profiles along the focal plane are compared. (**c**) Focal length and depth of focus of OPtIC microlenses are obtained using intensity profile along the optical axis. (**d**) Transmission spectra is shown. Extraordinary light transmission effect leads to a transmission peak at 650 nm.

A critically important device parameter is the nanofluidic channel (center aperture) diameter, which controls the size exclusion range in addition to the focusing behavior of the OPtIC microlens^13^. The enlarged center aperture *d*_*c*_ must be incorporated without deteriorating the focusing characteristics and the desired size selectivity. In Fig. 2a, intensity profiles of OPtIC microlenses with varying center aperture diameters are shown for an incident light beam at *λ* = 633 nm. The intensity profiles of a NHA without a center aperture (*d*_*c*_ = 0), a uniform NHA (*d*_*c*_ = 150 nm) and a NHA with an enlarged center aperture (*d*_*c*_ = 500 nm) reveals that the enlarged center aperture has negligible effect on the focusing behavior. Even for significantly larger center aperture dimensions (*d*_*c*_ = 800 nm), OPtIC microlens manages to focus light to a tight spot, albeit with a lower efficiency (degraded focusing characteristics). The checkerboard-like intensity profile close to the microlens surface (see Supplementary, Fig. S1) is due to the diffractive self-imaging of smaller size nanoholes around the center aperture, an effect known as plasmonic Talbot effect^13,24^. The enhanced light intensity regions around the larger diameter center openings (*d*_*c*_ = 500 nm and 800 nm) is associated to the diffractive transmission of light through the enlarged aperture. As shown in Fig. 2b, for large enough openings (*d*_*c*_ ≥ 500 nm), the light intensities in these diffractive transmission regions are comparable to or higher than those at the focal point. However, in contrast to the focal point, fluidic drag forces (**F**_**d**_ ∝ **v**) are much stronger than the optical scattering forces (F_d_ ≫ F_s_) in these diffractive transmission regions. Our analysis shows nearly three orders of magnitude enhanced fluidic flow velocities close to the center aperture as the fluidic flow squeeze through the narrow center aperture with 500 nm diameter (Fig. 1b). Hence, small size and lower refractive index particles that are filtered through the focal point region can follow the fluidic flow lines to the other side of the OPtIC microlens without hinderance in the diffractive transmission regions close to center aperture. Therefore, in the following, we optimized our OPtIC microlens design for the focal point where comparable strength optical scattering, thermo-plasmonic convection and fluidic drag forces can be readily created for selective sorting purposes.

Enlarged center aperture minimally affects the focal distance *f*_*D*_, as shown in Fig. 2b. The focal distance *f*_*D*_ is 5.32 μm for *d*_*c*_ = 0, 150 nm, and 500 nm, whereas it only slightly increases to 5.56 μm for *d*_*c*_ = 800 nm. Similarly, a wider focal spot size at *z* = *f*_*D*_ is observed for microlenses with larger center apertures (Fig. 2c). For microlenses with *d*_*c*_ = 0 and 150 nm, spot sizes are almost identical with a full-width at half maximum (FWHM) spread of *w* = 1.12 μm. For *d*_*c*_ ≥ 500 nm, *w* increases with increasing center aperture opening; for *d*_*c*_ = 500 nm and 800 nm, we find that the spot sizes are *w* = 1.24 μm and 1.80 μm, respectively. Focusing characteristics of OPtIC microlenses are mainly controlled by the smaller diameter nanohole array created around the center enlarged aperture. Transmission spectra obtained using a field monitor is shown in Fig. 2d. EOT effect occurs at wavelengths that satisfy the Bragg condition: $\overrightarrow{G}=i{\overrightarrow{G}}_{x}+j{\overrightarrow{G}}_{y}$, where (*i, j*) are the integer values for the corresponding grating order^17,19^. In Fig. 2d, the grating coupled (1,0) resonance transmission peak is observed at 650 nm (FWHM ~ 55 nm), confirming that light focusing is a result of interference of in-phase photons emerging from the periodic small size nanohole array^13^. For center apertures with diameters up to *d*_*c*_ = 500 nm (Fig. 2d, yellow curve), transmission spectra show minimal deviation from a uniform NHA microlens without a center aperture (*d*_*c*_ = 0)^13^, albeit with some broadband background transmission. On the other hand, for significantly larger center openings (*d*_*c*_ = 800 nm), non-resonant light transmission through the center aperture increases, leading to an increased background signal and broadening of the resonant transmission peak (Fig. 2d, violet curve). Based on the focusing behavior, attainable fluidic flow velocities at the focal point, and transmission spectra discussed above, we determined that an OPtIC microlens consisting of 9 × 9 array of nanoholes (diameter of *d* = 150 nm and a periodicity of *a*_0_ = 380 nm) with a center aperture of *d*_*c*_ = 500 nm is ideal for sorting of small size particles, such as exosomes. This microlens design is used in the rest of this paper.

**Figure 3:**
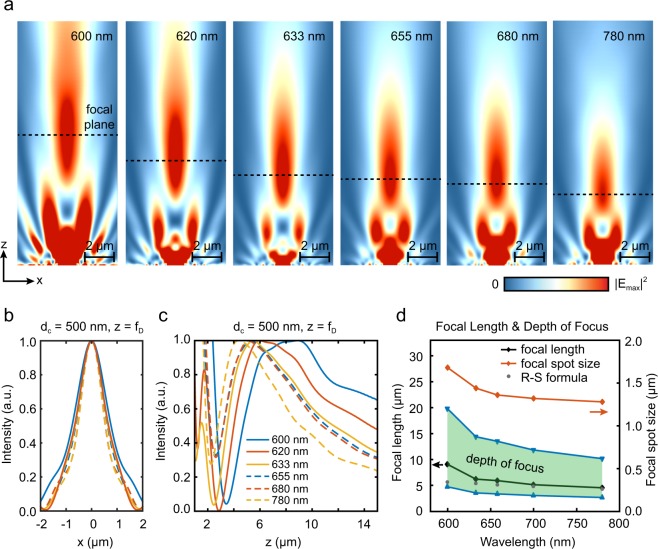
(**a**) Beam profile is shown for OPtIC microlens with 500 nm center aperture as function of incident light wavelength. (**b**) Field profiles along the focal plane are compared. (**c**) Focal length and depth of focus of OPtIC microlenses are obtained using the intensity profile along the optical axis. (**d**) Focal length, depth of focus and focal spot size are shown as a function of wavelength. Strong agreement with the values obtained from Rayleigh-Sommerfeld formula (grey dots) is observed.

High-intensity light sources, such as light emitting diodes (LEDs), are preferable to lasers for label-free size-based sorting applications. In this respect, NHA microlenses providing a focusing capability with minimal chromatic aberration are advantageous^13^. We analyzed the effect of center nanofluidic channel on the broadband focusing characteristics of our plasmofluidic microlens for a wavelength range spanning from 600 nm to 780 nm. Figure 3a shows that *f*_*D*_ decreases monotonically with increasing wavelength^13^. However, a particularly small focal length variation *Δz* ≈ 200 nm is observed for the wavelength range 620 nm < *λ* < 680 nm corresponding to the (1,0) resonant transmission (EOT) peak. Outside the EOT spectral window, a significantly longer focal length is found at the transmission minimum *λ* = 600 nm (Fig. 3a) corresponding to the Wood’s anomaly^20,25^. We calculated focal spot size (*w*) along the focal plane denoted by the horizontal dashed lines in Fig. 3a. Within the (1,0) resonant transmission peak, only a slight difference is observed for the focal spot size. As shown in Fig. 3b, *w* is 1.08 μm, 1.12 μm, 1.24 μm and 1.28 μm at *λ* = 620 nm, 633 nm, 655 nm and 680 nm, respectively. Similarly, light intensity profile along the OA presents minimal variations within the 620 nm < *λ* < 680 nm wavelength range (Fig. 3c). Hence, our analysis confirms that the (1,0) resonance transmission (EOT) peak provides a focusing behavior that is unaltered over a sufficiently-broad range of wavelengths.

The focal length of a finite-size opening that has identical dimensions to our plasmofluidic microlens can be calculated using the Rayleigh-Sommerfeld (R-S) formula^13,26,27^:

1 $$\frac{{\rm{d}}I}{{\rm{d}}z}=-\,2{I}_{0}\frac{\pi {\rho }^{2}}{\lambda {z}^{2}}\,\sin (\frac{\pi {\rho }^{2}n}{\lambda z})=0$$

where *z* is the distance above the lens, *I* and *I*_*0*_ are intensity and its peak values, respectively, whereas *ρ* is the radius of the opening and *n* is the refractive index of the surrounding medium. Our analysis shows that focal length *f*_*D*_ of our plasmofluidic microlens (Fig. 3d, black curve) is in good agreement with the values obtained from R-S formula (Fig. 3d, grey dots) for the wavelength range 620 nm < *λ* < 680 nm. Depth of focus (DoF) of our plasmofluidic microlens (shaded area bounded by the dashed lines) is shown in Fig. 3d. Even though the periodicity of nanohole array is broken with the incorporation of a nanofluidic channel (*d*_*c*_ = 500 nm) at the center, our analysis confirms that our plasmofludic microlens provides a well-defined focusing behavior with minimal chromatic aberration for the wavelength range of 620 nm <*λ* < 680 nm.

### Optical scattering, thermo-plasmonic and fluidic drag forces

Optical scattering forces acting on a bioparticle can be decomposed to two orthogonal components: a radial gradient force (**F**_**g**_) directed towards the beam axis and optical scattering force (**F**_**s**_) in the direction of beam propagation against the fluidic flow. These forces can be expressed as^14^

2 $${F}_{s,g}=\frac{2{n}_{m}P}{c}{Q}_{s,g}$$

where *P* is the power of the incident light, *n*_*m*_ is the refractive index of the medium, *c* is the speed of light, and *Q*_*s,g*_ is a dimensionless parameter defined for the scattering (*s*) and gradient (*g*) forces. *Q*_*s,g*_ represents the efficiency of optical pressure transfer as a result of the light reflection/refraction at material interfaces. For simple beam profiles and symmetric geometries (i.e., a mildly focused Gaussian beam acting on a spherical particle), it is possible to calculate *Q*_*s,g*_ analytically. For more complex beam profiles and small size particles with a diameter *a* < 1 μm, instead of the ray-optics model, optical forces must be calculated using Maxwell stress tensor (MST)^28,29^:

3 $${T}_{ij}=\varepsilon {E}_{i}{E}_{j}^{\ast }+\mu {H}_{i}{H}_{j}^{\ast }-\frac{1}{2}{\delta }_{ij}(\varepsilon |{\bf{E}}{|}^{2}+\mu |{\bf{H}}{|}^{2})$$

where **E** and **H** are the electric and magnetic field vectors, *ε* and *μ* are the electric permittivity and magnetic permeability of the medium, and *δ*_*ij*_ is the Kronecker delta. Using FDTD simulations, MST allows us to obtain scattering and gradient forces acting on a particle for an arbitrarily shaped electromagnetic (EM) field distribution. Assuming a bounding box, small enough to contain only the particle of interest, the net optical force on the particle can be calculated as^29^

4 $$F={\oint }_{S}\sum _{j}\frac{1}{2}\mathrm{Re}({T}_{ij}{\hat{n}}_{j})$$

where *S* is the surface of the bounding box and ${\hat{n}}_{j}$ is a unit vector along one of the principal axes.

For light intensities used here, electromagnetic heating of the OPtIC microlens can lead to large enough local temperature gradients inducing a buoyancy-driven convective flow away from microlens surface^30,31^. A comprehensive discussion of heat induced fluid dynamics can be found in elsewhere^32,33^. Here, contribution of thermo-plasmonic effects is incorporated using finite element method (FEM). We first solve the electromagnetic wave equation for the electric field ***E*** around the OPtIC microlens with a 500 nm-diameter center aperture^34^,

5 $${\rm{\nabla }}\times ({\rm{\nabla }}\times {\bf{E}})-{k}_{0}^{2}\varepsilon ({\bf{r}}){\bf{E}}=0$$

where *k*_0_ = 2π/*λ* is the free-space wave number, and *ε*(***r***) the position-dependent complex dielectric permittivity at the incident light wavelength. The calculated electric field distribution is used to obtain the heat source density *q*(**r**) = 0.5Re[**J** ⋅ E*], where **J** is the induced current density in the microlens^34^ and the total heat power ${\rm{Q}}=\,\iiint q({\bf{r}})dv$. We then used coupled steady-state heat transfer and incompressible Navier-Stokes relations to calculate the temperature and thermo-plasmonic velocity field distributions

6 $${\rm{\nabla }}\cdot [\rho {c}_{p}T({\bf{r}}){\bf{u}}({\bf{r}})-\kappa {\rm{\nabla }}T({\bf{r}})]=Q({\bf{r}})$$

7 $${\rho }_{0}[{\bf{u}}({\bf{r}})\cdot {\rm{\nabla }}]{\bf{u}}({\bf{r}})+{\rm{\nabla }}p({\bf{r}})-\eta {{\rm{\nabla }}}^{2}{\bf{u}}({\bf{r}})={\bf{F}}$$

where ***∇ ⋅ u*** = 0, *T*(***r***), ***u***(***r***), and *p*(***r***) are the spatial temperature, fluid velocity, and pressure distributions, respectively. The material parameters for thermal conductivity *κ*, density *ρ*, specific heat capacity *c*_*p*_, and kinematic viscosity *η* are taken from ref. ^33^. The steady-state temperature distribution on the OPtIC microlens surface under 20 mW illumination at 633 nm is shown in Fig. S2a. Perpendicularly incident light transmitting diffractively through the center aperture is weakly coupled to the plasmonic excitations and dissipated within the metal film. Hence, a relatively low temperature difference from the ambient is observed within the center aperture region. Significantly higher local temperatures above the ambient are established outside the center aperture region due to non-radiative decaying of SPPs excited on gold surface. Due to limited thermal conductivity, the generated heat dissipates slowly into the solution medium, establishing a convective current away from the OPtIC microlens surface (Fig. S2b). We incorporated the contribution of this heat-induced convective flow in particle motion using Boussinesq approximation^31,33,35^

8 $${{\bf{F}}}_{{\rm{t}}{\rm{p}}}=g{\rho }_{0}\beta (T)[T({\bf{r}})-{T}_{0}]\hat{z}$$

where ***F***_tp_ is the imposed volume force, *g*, *ρ*_0_, and *β* are the gravitational constant, water density, and thermal expansion coefficient of water, respectively.

The fluidic drag forces **F**_**d**_ acting on nanoparticles are calculated using Stoke’s relation

9 $${{\bf{F}}}_{{\bf{d}}}=-\,6\pi \eta R{\bf{v}}$$

where η is the viscosity of the liquid medium, **v** is the velocity of the particle relative to the liquid medium and *R* is the particle radius. Contribution of radial fluidic drag forces (**F**_**d,r**_**)** in focusing particles along the OA is separately discussed in our analysis below.

### Label-Free Sorting of Exosome Size Bioparticles

**Figure 4:**
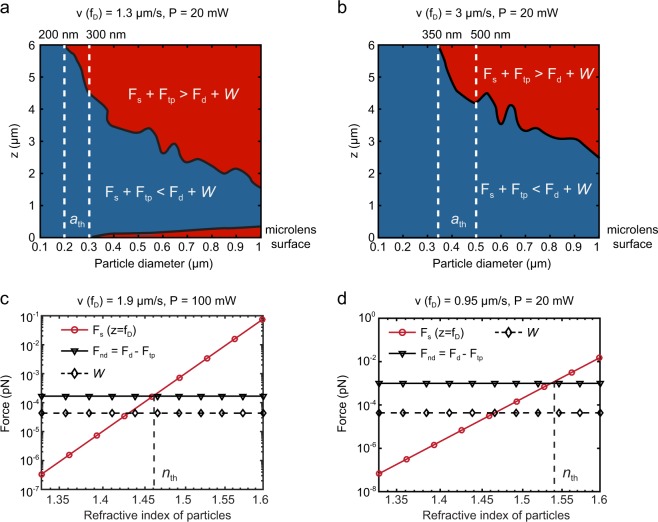
The net force (**F**_**net**_ = **F**_**s**_** + F**_**tp**_ **+ F**_**d**_ **+ W**) acting on submicron particles (a = 100 nm − 1 m) along the optical axis (z = 0–6 m) for flow conditions corresponding to (**a**) 1.3 μm/s and (**b**) 3 μm/s flow rates at the focal point. We assumed that 20 mW incident light (*λ* = 633 nm) and a fluidic flow velocity of 1.3 μm/s is used. Optical scattering (**F**_**s**_), net fluidic drag (**F**_**d**_ **+ F**_**tp**_), gravitational (***W***) forces acting on a 200 nm exosomes-like bioparticle are shown as a function of particle refractive index (*n*_*e*_) for (**c**) 1.9 μm/s flow rate and 100 mW incident power and (**d**) 0.95 μm/s flow rate and 20 mW incident power. The dashed vertical lines indicate the threshold particle refractive index (*n*_th_) for which the net force acting the particles vanishes. Here, the lines are the first-order polynomial curve fits.

The robust size selective separation capability of our OPtIC platform is realized by harnessing the varying strength of optical scattering (**F**_**s**_), thermo-plasmonic convection (**F**_**tp**_), fluidic drag (**F**_**d**_) and gravitational (***W***) forces based on size, refractive index and mass density of the bioparticles. The net forces (**F**_**net**_ = **F**_**s**_** + F**_**tp**_ **+ F**_**d**_ **+** ***W***) acting on submicron particles (a = 100 nm − 1 μm) at different locations along the OA (*z* = 0–6 μm) are shown in Fig. 4a,b. Here, a refractive index of 1.55 and a mass density of 1.05 g/cm^3^ are assumed for the interrogated bioparticles. In Fig. 4a,b, the blue (red) region corresponds to physical conditions (particle sizes and spatial locations along the OA) leading to a net force towards (away from) the center aperture. For particles with a small diameter (a_th_ < 200 nm), when 20 mW incident light (*λ* = 633 nm) and a fluidic flow velocity of 1.3 μm/s is used, optical scattering and thermo-plasmonic convention forces are weaker than the fluidic drag and gravitational forces (F_d_ + *W* > F_s_ + F_tp_) at all locations along the OA (Fig. 4a, the vertical line on left). Hence, small diameter particles (a_th_ < 200 nm) can readily follow the fluidic flow lines along the focal point and the high-intensity regions close to the center aperture. These particles (a_th_ < 200 nm) make their way through the center aperture to the other side of the suspended OPtIC microlens and get separated from the mixture in the top channel (negative depletion). Under the same operating conditions, larger diameter particles (a > 200 nm) experience stronger optical scattering and thermo-plasmonic convection forces that can push them against the fluidic flow lines  (F_d_ + *W* < F_s_ + F_tp_). These larger bioparticles (a_th_ > 200 nm) are retained in the top channel (Fig. 4a). Threshold bioparticle diameter *a*_th_ used for the size based fractionation can be readily tuned to a desired value by changing the light intensity and the fluidic flow velocity. In Fig. 4b, we show that the increased fluidic flow (velocity = 3.0 μm/s) shifts the threshold diameter (a_th_) for size based sorting to 350 nm. By fine-tuning the relative contributions of the optical scattering, thermo-plasmonic convection and fluidic drag forces, microvesicles (up to 500 nm in diameter) can be selectively separated from larger bioparticles.

Minor differences in internal structure and chemical composition of bioparticles lead to effective refractive index variations that can be exploited for selective separation^4,7^. A remarkable example of this is a recent study demonstrating that cells with single gene modifications can be distinguished based on their differences in chemical makeup using optical chromatography^3^. Based on the same physical principles, our plasmofluidic platform utilizing optical scattering, thermo-plasmonic convection and fluidic drag forces provides a highly sensitive refractive index-based bioparticle separation capability. In Fig. 4c, optical scattering **F**_**s**_ (red line) and net fluidic drag (black line) **F**_**d**_ + **F**_**tp**_ forces are compared at the focal point (*f*_*D*_ = 5.32 μm, *λ* = 633 nm, P = 100 mW) as a function of effective refractive index (*n*_*e*_) of bioparticles with a diameter of *a* = 200 nm. Gravitational forces (***W***) acting on these particles are also shown (black dashed line). Here, the optical scattering forces **F**_**s**_ (red curve) are calculated for an effective refractive index range spanning from *n*_*e*_ = 1.33 (water) to *n*_*e*_ = 1.6 (i.e., polystyrene particles). **F**_**s**_ increases with increasing *n*_*e*_ and balances the counter acting the net fluidic resistance force **F**_**d**_ + **F**_**tp**_ for *n*_*e*_ = 1.46 (i.e., phospholipids and proteins) at a flow rate of v(f_D_) = 1.9 μm/s. For nanoparticles that have refractive indices lower than *n*_*e*_ < 1.46, the fluidic drag forces are strong enough to carry them against the optical scattering forces through the enlarged center aperture to the chamber below the OPtIC microlens and separate them from a heterogenous mixture. This allows sorting of lower refractive index (*n*_*e*_ < 1.46) particles from higher refractive index (*n*_*e*_ > 1.46) ones. A particularly prominent observation is the significantly diminished optical scattering forces (~4 aN) obtained for the lower refractive index (*n*_*e*_ ≈ 1.37–1.39) particles, which is two order of magnitude weaker than the optical scattering forces (~400 aN) acting on higher refractive index particles with *n*_*e*_ ≈ 1.46 under same illumination conditions. Exosomes consisting of a thin phospholipid membrane enclosing a water load have lower effective refractive indices (*n*_*exosome*_ ≈ 1.37–1.39)^36–38^ that are closer to that of water (*n*_*water*_ ≈ 1.33). Virions, on the other hand, are a tight assembly of nucleid acids, proteins, and lipids; therefore have higher refractive indices (*n*_*virus*_ ≈ 1.48)^39^. Accordingly, our platform enables us to use this refractive index difference to separate exosomes from similar size virions (Fig. 4c). Similar to the size-based separation (Fig. 4a,b), the threshold refractive index can be tuned to a desired value by adjusting the fluidic flow rate and light power (Fig. 4d). As demonstrated by Fig. 4d, at a relatively small flow rate (0.95 μm/s) and incident power (20 mW), the net force (**F**_**s**_ + **F**_**tp**_ + ***W*** + **F**_**d**_) acting on the 200 nm particles vanishes for particles with higher refractive index (*n*_*e*_ ≈ 1.54) than those in Fig. 4c (*n*_*e*_ ≈ 1.46). Furthermore, multiple OPtIC microlenses integrated on a single planar chip can be serially implemented to achieve differential sorting using an initial size-based fractionation and a subsequently separation based on differences in bioparticle chemical makeup (refractive index).

### Radial focusing of the bioparticles

**Figure 5:**
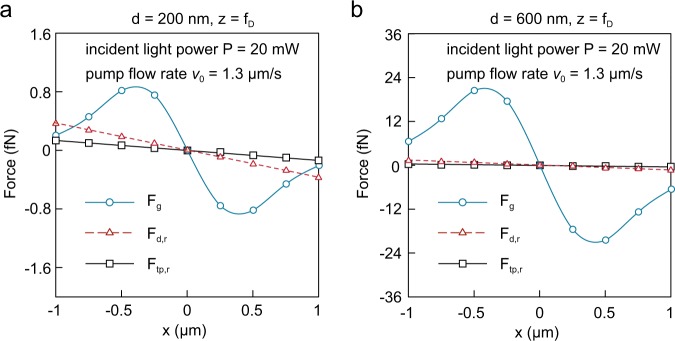
Optical gradient (**F**_**g**_), radial fluidic drag (**F**_**d,r**_), and radial thermo-induced fluidic drag (**F**_**tp,r**_) forces align particle trajectories with OA (x = 0). Force acting on particles are compared for particles with (**a**) a = 200 nm and (**b**) a = 600 nm diameter. Here, the curves are sinusoidal fits while the lines are the first-order polynomial curve fits.

It is predicted that the instrumental fluctuations associated to variations in fluidic flow velocities can cause spatial dispersion of particles, deteriorating the size-based retention capability of conventional optical chromatography approaches^6^. Instead of mildly focused Gaussian beams, our OPtIC microlenses use strongly focused light that can create large optical gradient forces **F**_**g**_ in radial direction. As shown in Fig. 5b,c for 200 nm and 600 nm diameter particles, in addition to the optical gradient forces **F**_**g**_ (blue curve), the spatial dispersion of particles in our platform is inherently minimized by the fluidic drag **F**_**d,r**_ (black curve) and thermo-plasmonic convection **F**_**tp,r**_ (red curve) forces working together to align particles along the OA (r = 0). Here, an incident light (*λ* = 633 nm) power of 20 mW and a fluidic velocity of 1.3 μm/s are assumed. For distances up to ±1 μm away from the OA, the optical gradient (**F**_**g**_), thermo-plasmonic convective flow (**F**_**tp,r**_) and fluidic drag (**F**_**d,r**_) forces are reminiscent of a restoring force of a spring, which tends to align the particles at equilibrium position (along the OA). Another important observation is the relative strength of the optical gradient forces **F**_**g**_ with respect to those of the radial fluidic drag forces **F**_**d,r**_. Although particles are initially brought towards OPtIC microlens through the fluidic flow, once they are close to high intensity focal point region, their relative alignment along the OA is mainly ensured by the optical gradient force. Therefore, our plasmofluidic optical chromatography technique provides a self-collimating mechanism for particle trajectories that is tolerant of the perturbations in fluidic flow rates.

## Discussion

Here, we introduced a facile on-chip platform enabling optical sorting of nano-bioparticles based on size and chemical composition (refractive index). Unlike existing optical chromatography techniques that require complicated instrumentation (lasers, objectives, precise stages, etc.) for optofluidic alignment and optical scattering force creation^2,5,10^, our platform achieves objective-free focusing of collimated broadband light and self-alignment of counteracting optical scattering and fluidic drag forces along the optical axis. To accomplish this, we developed a subwavelength thick optofluidic plasmonic (OPtIC) microlens merging focusing capabilities of NHA plasmonic microlenses^13,21^ with nanofluidics. Our OPtIC microlens effortlessly realizes precise alignment of nanoparticles along the optical axis using radially focusing fluidic drag and optical gradient forces. We demonstrated that our plasmofluidic platform facilitates selective separation of nanoparticles below a threshold diameter, which can be tuned to a desired value using fluidic flow rate and the incident light power. Furthermore, we showed that our platform offers a robust separation capability for same size nanoparticles using differences in their chemical composition (refractive index). A major limitation in flow cytometry measurements is the diminished optical signals obtained from small size and lower refractive index nano-bioparticles, such as exosomes^37^. Even with specialized flow cytometers equipped with high sensitivity detectors, the smallest detectable vesicles are typically larger than ~200 nm in diameter^37,40^. Hence, vast majority of exosomes are overlooked in flow cytometry measurements, which is the most commonly used optical method in clinical and research laboratories. In contrast, our optofluidic platform employs a negative depletion mechanism enabling selective enrichment of exosomes in a readily manner by removing the larger size and higher refractive index particles using optical radiation pressure. Furthermore, our platform could be used for quantification of exosomes passing through the center nanohole aperture through Coulter principle^41^. Two electrodes added on the opposite sides of the OPtIC microlens can detect brief changes (pulses) in the current that flows through the aperture, as the enriched exosomes transverse the enlarged center aperture. Previous studies have shown that resistive pulse sensing (RPS) technologies based on Coulter principle in aperture format are capable of detecting bioparticles smaller than 100 nm^42^. However, clogging of openings due to the larger particles has been a practical limitation when heterogenous samples are analyzed^37^. Our OPtIC microlens keeping larger diameter particles away from the nanoaperture opening using optical scattering forces, when combined with RPS, could overcome these limitations and be used for selective sorting and detection of exosomes from heterogenous samples. To achieve extended periods of operation and high-volume processing, one can incorporate a lateral microcross flow^43^ periodically removing the particle microfumes that build up above the focal point of the OPtIC microlens.

## Supplementary information

Supplementary Information
