# Supplementary material for: Plasmofluidic Microlenses for Label-Free Optical Sorting of Exosomes
Source: Sci Rep. 2019 Jun 13;9:8593. doi: 10.1038/s41598-019-44801-3 (PMC6565621; doi:10.1038/s41598-019-44801-3)
Supplement: Supplementary file 1 — Supplementary Information [file 41598_2019_44801_MOESM1_ESM.docx]

**SUPPLEMENTARY INFORMATION**

**Plasmofluidic Microlenses for Label-Free Optical Sorting of Exosomes**

Xiangchao Zhu^1^, Ahmet Cicek^2^, Yixiang Li^1^ and Ahmet Ali Yanik^1,3^

^1^Department of Electrical and Computer Engineering, University of California, Santa Cruz, CA 95064, USA.

^2^Department of Nanoscience and Nanotechnology, Burdur Mehmet Akif Ersoy University, Burdur 15030, Turkey.

^3^California Institute for Quantitative Biosciences (QB3), University of California, Santa Cruz, CA 95064, USA

**Corresponding Author:**

Prof. A. Ali Yanik

Department of Electrical & Computer Engineering

University of California, Santa Cruz

Santa Cruz, CA 95064

Email: [yanik@ucsc.edu](http://yanik@ucsc.edu)

**Near-Field Phase Map of Illuminated Microlens**


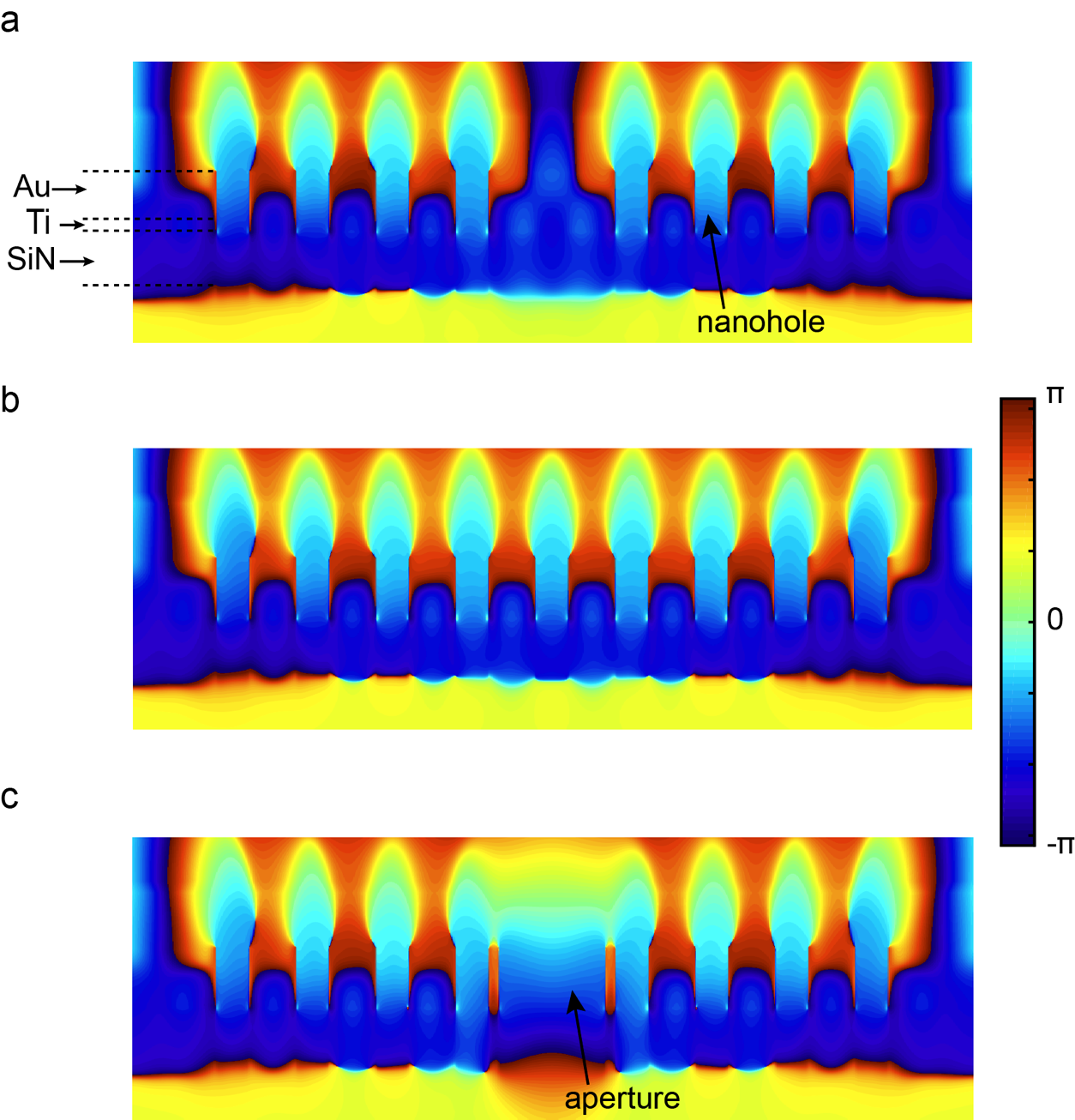


**Figure S1.** FDTD calculations of near-field phase map of electromagnetic waves emerging from the nanohole array (a) in the absence of the center nanohole (*d_c_* =150 nm), (b) in the presence of the center nanohole (*d_c_* =150 nm), (c) in the presence of the center enlarged aperture (*d_c_* =500 nm). Each nanohole transmits electromagnetic field with nearly equal amplitude and phase, resulting in in-phase interference of the electromagnetic waves. The absence of the center nanohole or diffractive transmission of light through the enlarged center aperture does not alter this checkerboard-like interference pattern.

**Thermo-Plasmonic Induced Convective Flow**


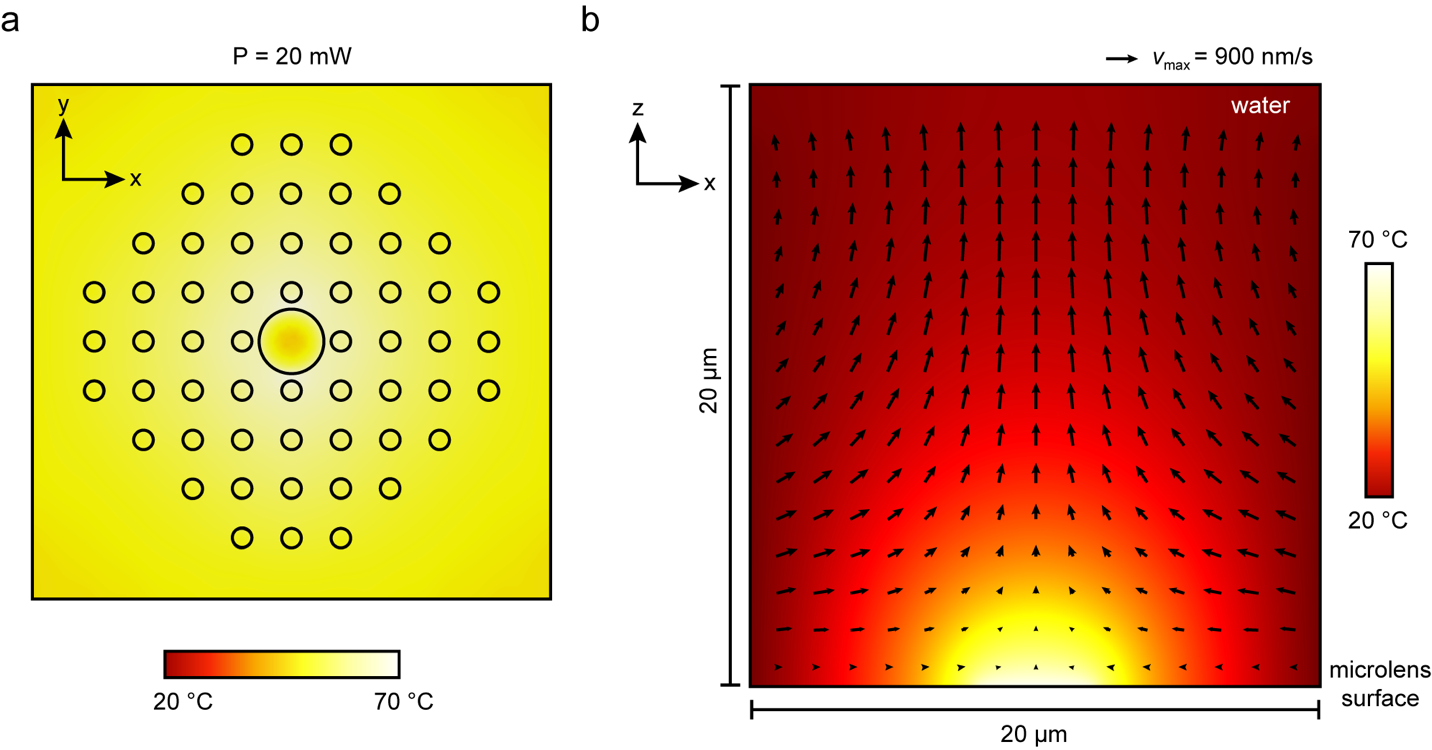


**Figure S2.** FEM calculations of electromagnetic heating of the plasmofluidic microlense induced temperature gradient and heat induced liquid convection flow. (a) Top view of the temperature distribution on the surface of the OPtIC microlens when illuminated with 20 mW light at 633 nm. (b) Thermo-plasmonic fluid convection current overlaid on the temperature distribution. The black reference arrow (*v*_max_) indicates the maximum velocity of 900 nm/s, the velocity at *f_D_* = 5.32 μm along the optical axis (OA) is approximately 360 nm/s.
